# 556. Evaluation of Hydrocortisone Use in Suspected Critical Illness-related Corticosteroid Insufficiency in Hypotensive Burn Patients

**DOI:** 10.1093/jbcr/irag033.296

**Published:** 2026-04-06

**Authors:** Riley Dean, Junlin Liao, Lucy Wibbenmeyer

**Affiliations:** University of Iowa, Iowa City, IA; The University of Iowa, Iowa City, IA; University of Iowa Health Care, Iowa City, IA

## Abstract

**Introduction:**

Critical illness-related corticosteroid insufficiency (CIRCI) is a syndrome in which critically ill patients exhibit relative adrenal insufficiency and tissue resistance to corticosteroids, resulting in vasogenic shock that is refractory to vasopressors. There are no current guidelines that support the use of corticosteroids to treat refractory shock in burn patients and research on its efficacy remains inconsistent and limited. We sought to review the use of corticosteroids in burn patients requiring vasopressors during their first week of treatment.

**Methods:**

Burn patients admitted from January 2015 to December 2025 requiring vasopressors during the first week constituted our study group. CIRCI was defined as treatment with corticosteroids. The burn registry and the electronic medical record were queried for the following variables: demographics, burn data, hospital course, and treatments. Outcome variables included length of stay (LOS), infections, graft loss, antibiotics days, vasopressor days, average glucose >200 days, and mortality. The two groups were compared with Students t-test and chi square where appropriate. A multivariant analysis including significant and related variables was performed to determine independent predictors of CIRCI and infection. Significance was determined at *p*<.05 level.

**Results:**

The study group consisted of 87 patients, 47 of whom received corticosteroids for a diagnosis of CIRCI and 40 who did not. All patients in the study group received vasopressors. Corticosteroids were started on average 2.85 ± 2 days (d) after admission. The average cortisol level in the CIRCI group was 13.7 vs 20.2 mcg/dL in the non-CIRCI group. The groups did not differ in demographics or etomidate use. The CIRCI group had higher TBSA (38.6 v 25.6%), more resuscitation (16.1 v 8.9 L), more vasopressor treatment (6.01 v 4.11 d), more ventilator days (20.9 v 8.1 d), more infections (66% v 34%) and longer LOS (52.24 v 28.22 d) all *p*<.05. On multivariant analysis, older age and more resuscitation fluids were related to the diagnosis of CIRCI, and corticosteroid administration and comorbidities were related to infection.

**Conclusions:**

Overall, over 50% of patients in hypotensive shock during their first week of burn were diagnosed with CIRCI and treated with corticosteroids. This group had larger TBSA and more intensive critical care needs. Steroid treatment and comorbidities were found to be independently associated with infections. More study is needed to determine the appropriate role of corticosteroid use in early burn treatment.

**Applicability of Research to Practice:**

The role of corticosteroids in the treatment of early hypotensive shock in burn care is unknown. Additional study of the use of corticosteroid use and sequela is needed.

**Funding for the study:**

N/A.

